# A novel *TEX11* mutation induces azoospermia: a case report of infertile brothers and literature review

**DOI:** 10.1186/s12881-018-0570-4

**Published:** 2018-04-16

**Authors:** Yanwei Sha, Liangkai Zheng, Zhiyong Ji, Libin Mei, Lu Ding, Shaobin Lin, Xu Wang, Xiaoyu Yang, Ping Li

**Affiliations:** 1 Reproductive Medicine Center, Xiamen Women’s and Children’s Health Hospital, Xiamen, 361003 Fujian Province China; 2 Department of Reproductive Medicine, First Affiliated Hospital of Nanjing Medical University, Nanjing, 210029 Jiangsu Province China

**Keywords:** *TEX11* mutation, Azoospermia, Infertility, Meiosis, Whole-exome sequencing

## Abstract

**Background:**

Testis-expressed gene 11 (*TEX11) is an X-linked gene and essential for meiotic recombination and chromosomal synapsis. TEX11 deficiency causes meiotic arrest and male infertility, and many TEX11 mutations have been found in azoospermic and infertile men.*

**Case presentation:**

*This study reported one novel TEX11 mutation* (2653G → T, in exon 29, GenBank accession number, NM_031276) *in two brothers with azoospermia. This mutation was firstly screened out by* whole-exome sequencing (WES) and further verified by amplifying and sequencing the specific exon 29. Surprisingly, the same exonic missense mutation (W856C) was observed in two brothers but not in their mother. Histological analysis of testicular biopsy from both brothers revealed meiotic arrest and no post-meiotic round spermatids and mature spermatozoa were observed in the seminiferous tubules. *TEX11* expression was observed strongly in spermatogonia and weakly in spermatocytes, but not in Sertoli cells and interstitial cells.

**Conclusions:**

We identified one novel *TEX11* mutation in two brothers and summarized the literature regarding *TEX11* mutations and male infertility. This study and previous literature indicate that *TEX11* mutations are closely associated with male infertility, especially azoospermia, although auxiliary clinical analyses are needed to figure out the causes of male infertility.

## Background

Infertility is defined as the inability to conceive after 12 months or more of unprotected sexual activity and is a major reproductive health problem [1, 2]. Infertility affects 10–15% of couples, and about 20–30% of infertility is caused by male factors. In humans, male infertility such as azoospermia or severe oligospermia is mostly characterized by decreased semen parameters [3]. The majority (~ 75%) of male patients with spermatogenic failure are idiopathic, and a genetic factor is often considered as the major causes [4, 5]. The most common strategies for the genetic diagnosis of male infertility are to screen for the presence of chromosomal aberrations, long arm of the Y chromosome (Yq) microdeletions, and gene mutations. In all cases of male infertility, karyotype abnormalities and Yq microdeletions are detected in ~ 5% and ~ 7.4% of patients, respectively [6]. In men with azoospermia, the prevalence of these two aberrations obviously increases to > 13% (chromosomal aberrations) and > 10% (Yq microdeletions) [6]. Yq microdeletions mainly occur in three or four “azoospermia factor” regions: AZFa, AZFb, AZFc, and AZFd (potential) [7]. It has been demonstrated that germ cell-specific genes are enriched in the mammalian X chromosome [5]. Many X-linked genes are expressed in the testis, and their functions in spermatogenesis have gradually been recognized in knock-out models and subjects with mutations. Three X-linked genes including testis-expressed gene 11 (*Tex11)*, TATA-box binding protein-associated factor 7 like (*Taf7l)* gene, and nuclear RNA export factor 2 (*Nxf2)* are known as important regulators of male fertility in mouse models [8–10]. The *Tex11* gene is conserved in vertebrates and encodes a 104-kDa protein with a tetratricopeptide repeat motif that mediates protein-protein interactions [11, 12]. *TEX11* can form distinct foci on meiotic chromosomes in spermatocytes and oocytes, thus it is considered as a meiosis-specific factor [13]. *TEX11* transcripts are exclusively expressed in the testis, and TEX11 protein is observed in the cytoplasm and nuclei of type B spermatogonia, with the highest level in zygotene spermatocytes, and a basal level in late pachytene spermatocytes [14, 15]. The abundant expression of TEX11 protein in type B spermatogonia and early spermatocytes supports that *TEX11* plays a key role in the early stage of germ cell development. In addition, Yang et al. have reported that male *TEX11*^−/−^ mice are infertile due to meiotic arrest [13]. Both Nxf2- and Taf7l-knockout mice exhibit reduced semen parameters and impaired motility [9, 10]. A kinase anchor protein 4 (*Akap4*)-deficient mice are infertile due to a poorly developed fibrous sheath and a short flagellum in the spermatozoa [16]. The androgen receptor gene on the long arm of the X chromosome has been found to play a role in meiosis and the conversion of spermatocytes to round spermatids during spermatogenesis [17]; while its mutation leads to androgen insensitivity syndrome and Kennedy syndrome, a neurodegenerative disorder with spermatogenesis abnormalities [18]. Moreover, mutations or partial deletions of some X-linked genes such as *AKAP3*, *AKAP4*, *NXF2*, *TAF7L*, *USP26*, and *TEX11* are linked to male subfertility or infertility [19–21].

In this literature review, we mainly discuss the dominant effects of *TEX11* on spermatogenesis and male infertility. Specific expression of TEX11 protein in spermatogonia and spermatocytes suggests that *TEX11* may play a role in the early stage of germ cell development [14, 15]. Interestingly, a recent study by Yu et al. has reported a negative effect of TEX11 on the proliferation of germ-cell-derived GC-1 and GC-2 cells [22]. They have found that TEX11 suppresses the phosphorylation of AKT and ERK signaling pathways through inhibiting ERβ binding to hematopoietic pre-B cell leukemia transcription factor-interacting protein [22]. Moreover, *Tex11* has been identified as the first X-encoded meiosis-specific factor in mice. In another study, Tang et al. have reported that *Tex11* is also exclusively expressed in germ cells in the adult pig testis. The expression of porcine *Tex11* is correlated with the onset of meiosis, and the expression pattern of *TEX11* homologs is highly conserved between pig and mouse [23]. Additionally, single nucleotide polymorphisms (SNPs) have a major impact in percentage of normal sperm, SNPs in *TEX11* gene showed close association with idiopathic male infertility [24–26]. After the generation of *Tex11*-deficient mice, the functions of the *Tex11* gene started to be uncovered. In 2008, Yang et al. generated *Tex11*-null mice by deleting 27 of the total 30 exons in the *Tex11* gene [13]. In *TEX11*-deficient mice, spermatogenesis is impaired due to chromosomal asynapsis at the pachytene stage and reduced crossover formation at the anaphase I stage [13]. *TEX11*-deficient spermatocytes mostly undergo apoptosis at the pachytene stage, while survived cells display chromosome nondisjunction at the first meiotic division, which causes cell death and male infertility [13]. Interestingly, another group generated a *Tex11* mutant strain by deleting exon 3, and found that the mutant males and females showed normal fertility, but the mutant males exhibited delayed repair of double-strand breaks (DSBs) in spermatocytes [14]. DSB repair and chromosome synapsis exert key roles in maintaining genetic integrity, while their malfunctions will cause various diseases such as infertility. In *TEX11* mutant (exon 3 deletion) mice, spermatocytes exhibited delayed repair of DSBs and decreased crossover formation [14]. Due to the unique hemizygous and transcriptional status of the X chromosome, it is well-recognized that the mutations in single-copy X-linked genes cannot be compensated by a corresponding wild-type allele [4]. Recently, X-linked *TEX11* mutations have been observed in infertile men [4, 21]. Yang et al. sequenced the amplicons covering the *TEX11* exons 2–30 and flanking intronic regions in a large cohort of infertile men with nonobstructive azoospermia as well as fertile controls. They observed a total of 40 different sequence variants in the introns and exons of *TEX11* [4]. Among them, 21 variants were singletons (only observed in one man, 18 variants in infertile men), while 19 variants were observed in 2 or more infertile men and/or fertile controls [5]. Moreover, another recent study screened for mutations in the *TEX11* open reading frame in 289 patients with azoospermia and 384 controls [21]. They identified the loss of three *TEX11* exons (exons 10–12) in two patients with azoospermia and five novel *TEX11* mutations (three splicing mutations and two missense mutations) in 7 of 289 (2.4%) men with azoospermia [21]. Therefore, the identification of mutations in X-linked genes essential for fertility may be important to figure out the underlying causes of male infertility, especially in men with azoospermia or severe oligospermia.

In the present study, we reported a case of two sterile brothers due to severe nonobstructive azoospermia and analysed the genetic causes by whole-exome sequencing (WES). In addition, we summarized the literature regarding *TEX11* mutations and male infertility.

## Case presentation

### Patients and medical examinations

This study was approved by the Ethics Committee of Xiamen Women’s and Children’s Health Hospital. Written consent was obtained from two brothers and their mother, and the family member of two fertile controls who died from car accident. Two infertile brothers attended the Reproductive Medicine Center of Xiamen Women’s and Children’s Health Hospital (Fujian Province, China) due to a history of primary infertility that had lasted for longer than 2 years. A questionnaire as well as standardized physical, clinical, and laboratory examinations were carried out to record details of their lifestyle, habits, occupation, marriage, family history, physical information (height and body weight), and chromosome and hormone levels (listed in Table 1). There was no history of genetic diseases or infertility in their family. Their parents had a nonconsanguineous marriage. The medical history of both spouses was unremarkable, and all parameters of the female medical check-up were normal. To verify their azoospermia status, three semen analyses (1 week interval) were carried out after 3 days of sexual abstinence, according to WHO guidelines [27]. No sperm was found in each round of analyses, so the two brothers were diagnosed as having azoospermia.

**Table 1:** The clinicopathological variables of two infertile brothers

	Patient 1 Value (normal range)	Patient 2 Value (normal range)
Age	30 years old	29 years old
Time of marriage	April 2012	August 2013
Height	166 cm	169 cm
Body weight	61 kg	66 kg
occupation	soldier	salesman
Testicular volume	~15 mL	~15 mL
Lateral spermatic vein	normal	normal
Chromosome	46, XY	46, XY
Y chromosome microdeletion	Not detected	Not detected
Follicle-stimulating hormone	5.92 mIU/mL (1.27–19.26)	6.25 mIU/mL (1.27–19.26)
Luteinizing hormone	4.33 mIU/mL (1.24–8.62)	5.27 mIU/mL (1.24–8.62)
Testosterone	3.06 ng/mL (1.75–7.81)	6.57 ng/mL (1.75–7.81)
Estradiol	22 pg/mL (10–60)	51 pg/mL (10–60)
Prolactin	23.43 ng/mL (2–18)	9.93 ng/mL (2–18)

### Cytogenetic and molecular genetic analyses

To screen the chromosomal status, cytogenetic chromosomal karyotype analysis and a fluorescent in situ hybridization (FISH) assay were conducted in two brothers and their mother using the peripheral blood cells. Karyotype and FISH analyses were carried out as described previously [28]. The VYSIS AneuVysion DNA Probe Kit (Abbott Laboratories. Abbott Park, IL, USA) was used (incorporating CEP probes for chromosomes 13 and 21 and LSI probes for chromosome X, Y and chromosome 18). A total of 20–100 metaphase cells were analyzed by the G-banding method according to ISCN 2013 guidelines [29], and the chromosome length consisted of approximately 450–550 sub-bands. FISH analysis was conducted with the combination of *SRY* and DYZ3 probes. Moreover, screening of Y chromosome deletions in two brothers were analyzed by a real-time PCR method as described previously [19]. According to the European Academy of Andrology and the European Molecular Genetics Quality Network guidelines, three selected sequence-tagged sites within specific AZFa, AZFb, and AZFc regions were chosen as targets. The *SRY* region was also examined. Four hydrolysis probes were designed to detect the four amplicons, respectively. The assay was carried out in a quadruplex reaction.

According to the results of the karyotype and FISH analyses, the karyotype of both brothers was normal (46, XY), and no gonadal mosaicism was observed although we observed gonadal mosaicism in the mother of both brothers. Moreover, we did not observe any Y chromosome microdeletions in either brothers.

To determine possible mutations causing azoospermia, whole-exome sequencing (WES) in two brothers and their mother was conducted as described previously [30]. Briefly, genomic DNA from two brothers’ semen and their mother’s blood cells was prepared in Illumina paired-end libraries and sequenced by using the Illumina HiSeq 2000 platform. The data were processed and analyzed, according to previous protocols [30]. To screen specific mutations, all variants of the genomewide data were compared to external publicly available databases including the 1000 Genomes Project (http://www.1000genomes.org) and other large-scale exome sequencing projects. Since *TEX11* is an X-linked gene, we did not examine the *TEX11* allele in the father of the two brothers.

**Fig. 1:**
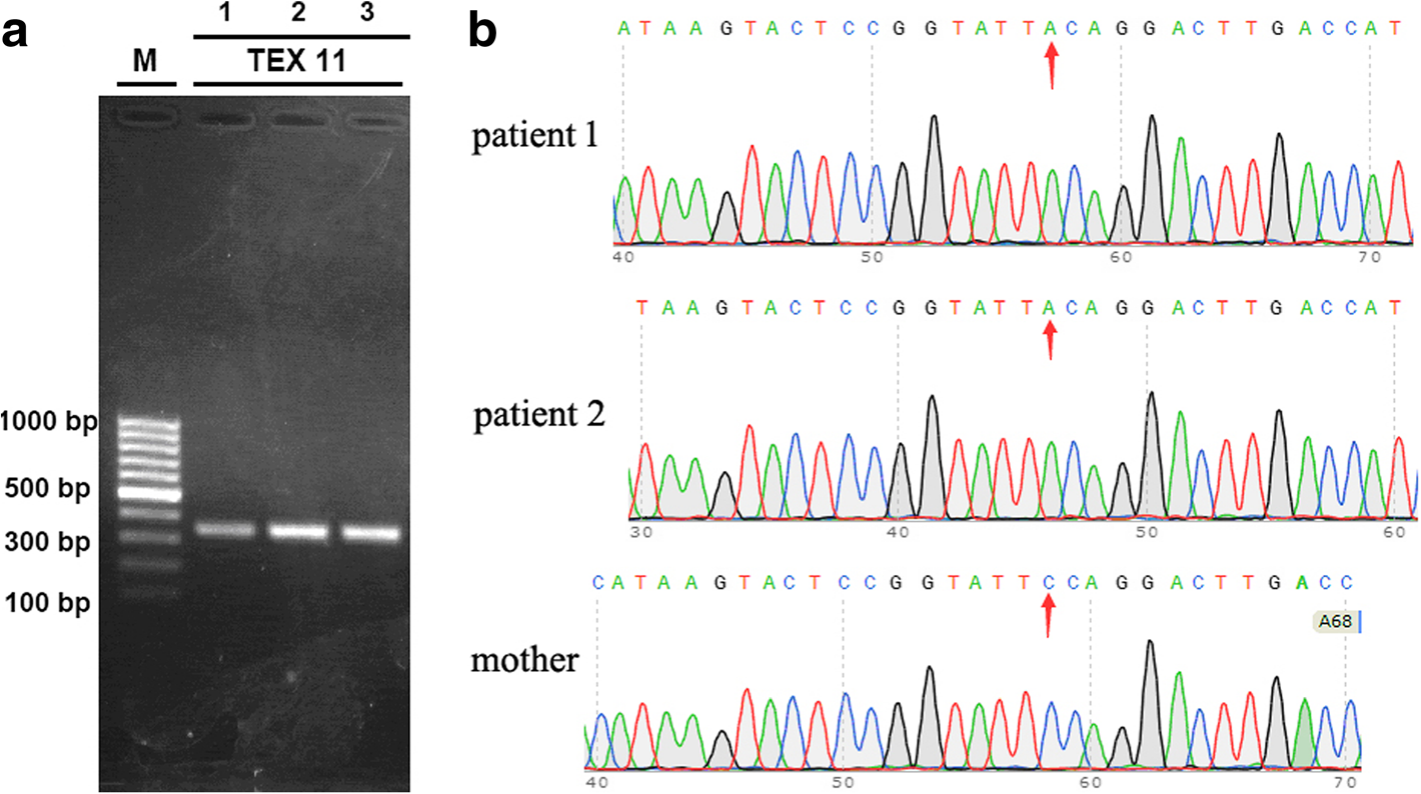
Identification of the *TEX11* mutation. **a** Amplification of *TEX11* exon 29 by PCR. Conventional end-point PCR was performed to amplify exon 29 of the *TEX11* gene from genomic DNA of two brothers (lanes 1 and 2) and their mother (lane 3). One clear and specific band at 100 bp was observed. **b** Mapping of the *TEX11* mutation. The PCR product sequences from the two brothers and their mother were verified by Sanger sequencing and aligned to human *TEX11* cDNA. Because Sanger sequencing was carried out using the reverse primer, the representative sequences were complementary to human *TEX11* cDNA (GenBank accession number, NM_031276). Accordingly, the mutation (C → A) in the map was indeed G → T in human *TEX11* cDNA

A mutation (2653G → T, GenBank accession number, NM_031276) in the exon 29 of the *TEX11* gene in the X chromosome was identified by WES in two brothers. However, this mutation was not observed in their mother’s WES. To verify the mutation identified by WES, exon 29 of the *TEX11* gene was amplified from genomic DNA of two brothers and their mother using conventional end-point polymerase chain reaction (PCR) and the following primers: forward, 5′-CTTGCTATGGAACATTCTACAG-3′; reverse, 5′-TGAAGGAGGTAAGGTGGTTA-3′. The PCR product with the appropriate size was observed in both brothers and their mother (Fig. 1a). The sequences of the PCR products were verified by Sanger sequencing. Consistent with the results of WES, the mutation (2653G → T) was verified in two brothers but not in their mother (Fig. 1b). Accordingly, the change of amino acids was determined to be W856C.

### Histological analysis

To determine the relationship between the histological change and azoospermia, testicular biopsies were performed in two brothers after their approval. Testicular tissues were fixed in 4% formaldehyde at 4 °C for 4 h, dehydrated in graded ethanol, embedded in paraffin, and cut into 4-μm-thick sections. To examine the testicular histology, the sections were deparaffinized, rehydrated in graded ethanol, and stained by hematoxylin and eosin (H&E). As a control of normal testicular histology, testicular sections from two fertile men who died from car accident and their body was donated by their family member to our hospital, were obtained from the Pathology Department of our hospital. The seminiferous tubules of fertile testes contained Sertoli cells and a full spectrum of spermatogenic cells including round spermatids and mature spermatozoa. To investigate the effect of *TEX11* mutation on the protein expression, we examined TEX11 protein expression in the testicular biopsies from two brothers and a normal testis by immunohistochemistry. Immunostaining of TEX11 was carried out using polyclonal goat-anti-human TEX11 antibody (ab99461, 1:100 dilution, Abcam, Cambridge, MA, USA) on a BenchMark XT automated immunohistochemistry/FISH slide staining system (Roche Diagnostics (Shanghai) Limited, Shanghai, China), according to the manufacturer’s instructions.

**Fig. 2:**
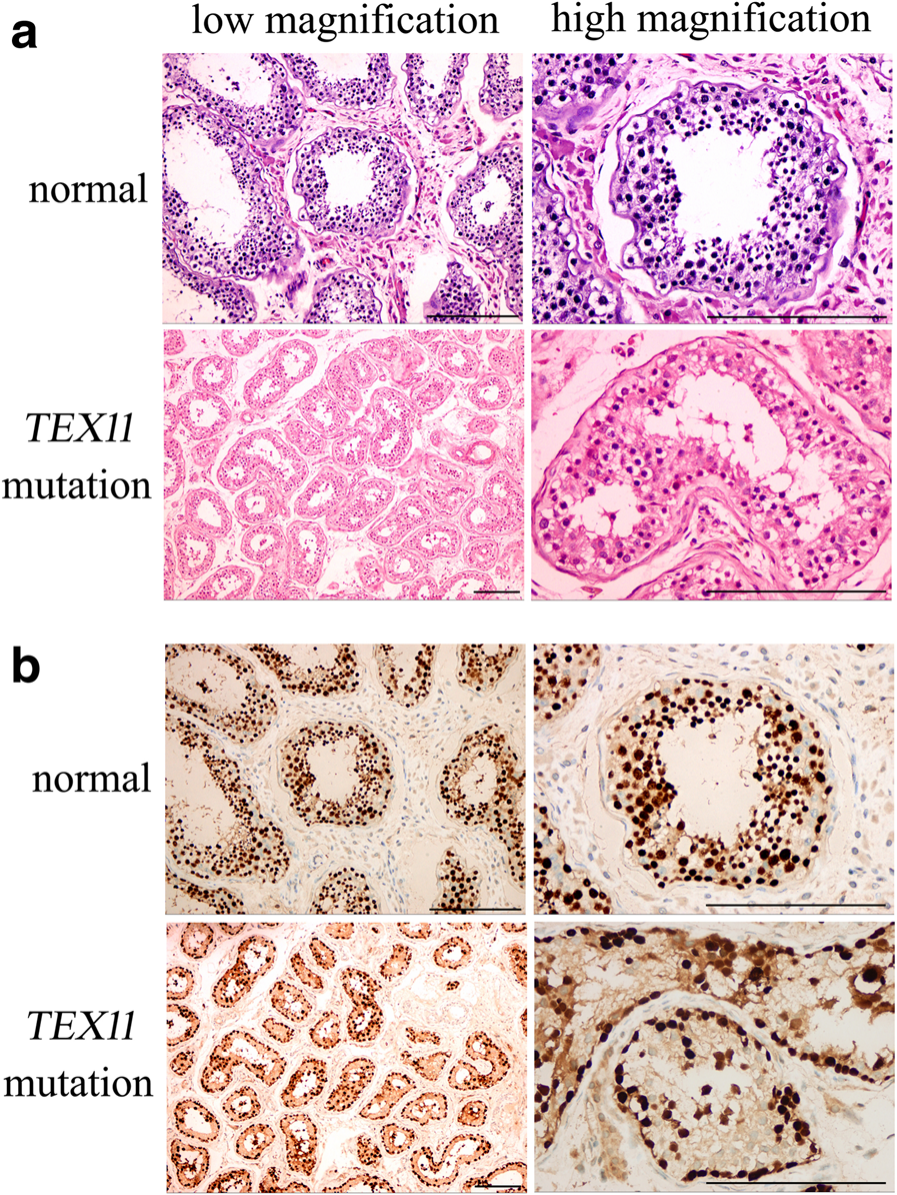
Histological examination and TEX11 staining in testicular biopsies. **a** Representative image of testicular histology from the older brother with azoospermia by hematoxylin and eosin staining. Testicular tissue from a healthy fertile man was used as a control. **b** Immunostaining of TEX11 in a testicular biopsy from the younger brother with azoospermia. TEX11 expression in testicular tissue from a healthy fertile man was used as a control. Scale bar = 100 μm

Compared with normal testicular histology, the testicular histology from two brothers showed a thicker basement membrane of seminiferous tubules and poorly developed spermatocytes (Fig. 2a). No post-meiotic round spermatids or mature spermatozoa were observed in the seminiferous tubules (Fig. 2a), which is consistent with the typical characteristics of azoospermia. In the normal testis, TEX11 protein was present in spermatogonia, spermatocytes, round spermatids, and mature spermatozoa in the seminiferous tubules, but it was absent in the surrounding somatic cells including the Sertoli cells and interstitial cells (Fig. 2b). However, in the testicular biopsies from the *TEX11*-mutated patients, TEX11 protein was observed strongly in spermatogonia and weakly in spermatocytes, while no TEX11 staining was found in Sertoli cells or interstitial cells (Fig. 2b).

## Discussion and conclusions

About 20–30% of infertility is caused by male factors [31]. The sex chromosomes (X and Y) in men play key roles in germ cell development. Both chromosomes contain a single copy of genes that are uniquely expressed in male spermatogenesis [20]. In the last decade, extensive efforts have been made to clarify the exact nature of male infertility; however, a large number of infertile men are diagnosed as having “idiopathic infertility”. It has been demonstrated that the common genetic causes for male azoospermia are Y chromosome deletion and chromosomal abnormalities such as Klinefelter syndrome (47, XXY karyotype), and these genetic causes are responsible for ~ 25% of spermatogenic failure in males [32, 33]. In the past decade, new technologies including array comparative genomic hybridization (ACGH), single nucleotide polymorphism (SNP) arrays, and next-generation sequencing have been used to detect infertility-related genetic defects [17, 20, 30]. A systematic genomic screening of mouse spermatogonia has demonstrated that the genes expressed specifically in male germ cells and located in the X chromosome play a critical role in early spermatogenesis [15].

Since *TEX11* is essential for meiotic recombination and chromosomal synapsis and *TEX11* deficiency causes meiotic arrest and male infertility, the identification of *TEX11* mutations has become attractive to determine the underlying causes of male infertility, especially in men with azoospermia. It has been found that *TEX11* mutations are diverse and present in various forms, such as missense and silent mutations, intronic alterations, frameshift mutations, and hemizygous deletions. Currently, 46 different *TEX11* mutations have been identified, including 24 in azoospermic men and 22 in fertile subjects [4, 21]. The incidence of the *TEX11* mutation in azoospermic men from Germany with a European descent was only 2.4% (7/289), while its prevalence was 14.5% (35/246) in American azoospermic males [4, 21]. It would be interesting to know whether this discrepancy is caused by the ethnicity. Two recent studies extensively investigated *TEX11* mutations in a large cohort of infertile/azoospermic men [4, 21]. Yatsenko et al. have identified six different *TEX11* mutations, including loss of exons 9–11 (607del237bp), three splicing mutations (405C → T, 748 + 1G → A, and 1793 + 1G → C), and two missense mutations (466A → G and 2047G → A) (Table 2) [21]. In addition, Yang et al. have carried out a more comprehensive sequencing analysis of *TEX11* exons and flanking introns in a large number of patients with nonobstructive azoospermia and fertile controls. They identified a total of 40 different sequence variants of *TEX11* in all subjects, but 22 different *TEX11* mutations (including three singletons) were observed in fertile controls, suggesting that these mutations are not linked to spermatogenic failure [4]. Eighteen of the 21 singletons were found in patients with azoospermia, which included five exonic missense mutations (W117R, V142I, Q172R, T244I, and V748A), two exonic silent mutations (405C → T and 2319 T → C), one exonic frameshift mutation, and 10 intronic mutations (Table 2). Some subjects exhibit multiple *TEX11* mutations. Interestingly, many intronic alterations such as −48G → A and + 42C → A and silent mutations like 405C → T and 2319 T → C cause meiotic arrest, while some exonic missense mutations (K115R, M152 V, E436K, and D832E) have been found in fertile men [4]. In the present study, we identified one novel exonic missense *TEX11* mutation (W856C) in two brothers but not their mother. Based on the testicular histology of two brothers, we observed a thicker basement membrane of the seminiferous tubules and poorly developed spermatocytes. No post-meiotic round spermatids or mature spermatozoa were observed in the seminiferous tubules, suggesting that this mutation will cause meiotic arrest. Moreover, Yatsenko et al. have observed that TEX11 expression is absent in the majority of seminiferous tubules but can be seen in rare tubules with remaining late spermatocytes and round spermatids [21]. In contrast, we found positive TEX11 staining in all seminiferous tubules, with strong expression in spermatogonia and weak expression in spermatocytes. This difference is probably due to different *TEX11* mutation sites or antibody specificity. Although the precise causes for most *TEX11* mutations remain unclear, Yang et al. have verified that one frameshift mutation of *TEX11* in an azoospermic man with meiotic arrest was inherited from his mother because his mother was heterozygous for this mutation and his brother was azoospermic [4]. Interestingly, we observed the same *TEX11* mutation in two brothers from a family without a history of infertility, whereas their mother had the wild-type allele of *TEX11*. The abundance of *TEX11* mutations increases the difficulty in identifying which mutations will cause male infertility.

**Table 2:** Mutations of *TEX11* detected in patients with azoospermia^*^

Position	Nucleotide change	Protein/RNA change	Spermatogenic failure	No. of patients	Ref.
Exon 6	405C→T	Silent mutation, A135spl d ^#^	Few sperm	1	21
Exon 7	466A→G	Missense mutation, M156V	No sperm	1	
Exons 9–11	607del237bp	203del79aa	Few sperm	2	
Intron 10	748+1G→A ^†^	L249spl d ^#^	No sperm	1	
Intron 21	1793+1G→C ^†^	R597spl d ^#^	No sperm	1	
Exon 24	2047G→A	Missense mutation, A683T	Few sperm	1	
Exon 6	349T→A	Missense mutation, W117R	No sperm	1	5
Exon 6	405C→T	Silent mutation	No sperm	1	
Exon 7	424G→A	Missense mutation, V142I	No sperm	1	
Exon 7	515A→G	Missense mutation, Q172R	No sperm	1	
Exon 10	731C→T	Missense mutation, T244I	No sperm	1	
Exon 16	1258Ins (TT)	Frameshift mutation; 1258GATG→TTGGTA	No sperm	1	
Exon 26	2243T→C	Missense mutation, V748A	No sperm	1	
Exon 27	2319T→C	Silent mutation	No sperm	1	
Intron 3	−17T→C ^†^	Intronic alteration	No sperm	1	
Intron 5	−48G→A ^†^	Intronic alteration	No sperm	1	
Intron 10	+42C→A ^†^	Intronic alteration	No sperm	1	
Intron 12	−28T→C ^†^	Intronic alteration	No sperm	1	
Intron 15	−64G→A ^†^	Intronic alteration	No sperm	1	
Intron 21	−1G→A ^†^	Alteration of splicing acceptor site	No sperm	1	
Intron 22	−37A→G ^†^	Intronic alteration	No sperm	1	
Intron 24	+119G→A ^†^	Intronic alteration	No sperm	1	
Intron 27	−55A→C ^†^	Intronic alteration	No sperm	1	
Intron 28	−44A→G ^†^	Intronic alteration	No sperm	1	
Exon 29	2568G→T	Missense mutation, W856C	No sperm	2	This study

^*^ TEX11 mutations are mapped to isoform 2 (GenBank accession number, NM_031276)

^#^ The term spl d represents the splicing donor site

^†^ +1 refers to the first base of a given intron, while -1 denotes the last base

In conclusion, we identified one novel *TEX11* mutation in two azoospermia brothers and summarized the literature regarding *TEX11* mutations and male infertility. *TEX11* mutations are closely related to male infertility, especially azoospermia. Auxiliary analyses such as testicular histology and family infertility history will also help to figure out the relationship between *TEX11* mutations and male infertility.

## Abbreviations

FISH: Fluorescent in situ hybridization
PCR: Polymerase chain reaction
WES: Whole-exome sequencing
